# Shear, Bulk, and Young’s Moduli of Clay/Polymer Nanocomposites Containing the Stacks of Intercalated Layers as Pseudoparticles

**DOI:** 10.1186/s11671-016-1703-3

**Published:** 2016-10-28

**Authors:** Yasser Zare

**Affiliations:** Young Researchers and Elites Club, Science and Research Branch, Islamic Azad University, Tehran, Iran

**Keywords:** Clay/polymer nanocomposites, Incomplete exfoliation, Pseudoparticles, Shear, bulk, and Young’s moduli

## Abstract

The pseudoparticles include the stacks of intercalated layers in the case of incomplete clay exfoliation in clay/polymer nanocomposites. In this article, the effects of pseudoparticle properties on the shear, bulk, and Young’s moduli of nanocomposites are studied using the Norris model. The properties of pseudoparticles are determined in some samples by the experimental data of Young’s modulus and the roles of pseudoparticles in the shear, bulk, and Young’s moduli of nanocomposites are discussed. The calculations show a good agreement with the experimental data when the pseudoparticles are taken into account in the samples. A low number of clay layers in the pseudoparticles present high moduli in nanocomposites. Moreover, the Poisson ratio and Young’s modulus of polymer matrix play different roles in the shear, bulk, and Young’s moduli of nanocomposites.

## Background

Only a little amount of clay can offer dramatic enhancements in stiffness, hardness, heat distortion temperature (HDT), and gas barrier properties of polymers [1–5]. Montmorillonite is mica-type silicate clay which consists of stacks of layers with only weak van der Waals force between the adjacent layers. Each single layer has a thickness of about 1 nm and lateral dimensions up to the micrometer scale. The microstructure of clay in clay/polymer nanocomposites (CPN) forms as phase separated, intercalated, or exfoliated [6–8]. If the clay stacks do not interact with the polymer chains, phase separation is occurred. In the case of intercalation, the polymer chains are inserted between the layers of the stacks and only the inter-layer spacing is expanded, but the layers still show a well-defined structure. However, the layers of clay are completely separated and the individual layers are distributed in the polymer matrix in the exfoliated structure.

The clay shows a significant Young’s modulus of about 180 GPa [9], but it cannot cause a considerable reinforcement in polymer nanocomposites in practice. The low stiffness of CPN significantly limits the marketable uses and many researchers have tried to solve this problem in recent years. It was reported that 7 wt.% of clay in organoclay (modified clay)/PP improves the Young’s modulus from 0.78 to 1.12 GPa [10]. Also, the Young’s modulus in PP/neat clay improves from 1.43 to 1.88 GPa by 5 wt.% of clay [11]. These examples show that the clay cannot produce the expected reinforcement in CPN.

The main reason for this phenomenon can be the imperfect exfoliation of clay layers in the polymer matrices which uncontrollably causes a complex structure with low reinforcement [12, 13]. Fornes and Paul [14] found that the intercalated layers in stacks decrease the reinforcing efficiency of clay in CPN compared to the exfoliated layers. The overall properties of CPN depend to the microstructure of clay such as the aspect ratio and dispersion quality of layers in polymer matrix. A complete exfoliation increases the aspect ratio and dispersion/distribution of layers which can cause a high level of interfacial involvement between polymer chains and clay layers. Moreover, the exfoliated layers efficiently alter the general characteristics of polymer matrix such as dynamics, crystallinity, and degradation temperature [15, 16]. Nevertheless, the complete exfoliation of clay layers in CPN was not reported in many samples and a mixed morphology of intercalated/exfoliated was commonly shown in CPN at the best condition.

**Fig. 1:**
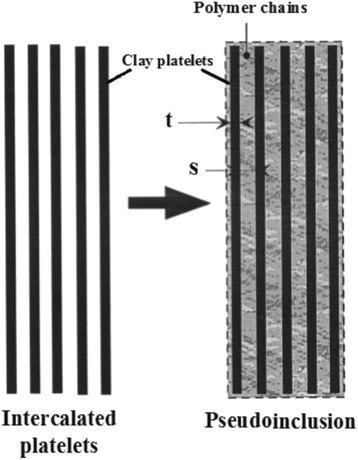
The pseudoparticles containing the stacks of intercalated layers

Since the incomplete exfoliation of clay layers has been observed in many samples, the researchers have tried to study the behavior of CPN such as modulus assuming the intercalated structure. Brune and Bicerano [17] assumed the pseudoparticles including the stacks of intercalated layers in which the polymer chains exist between the layers (Fig. 1).

They related the reduction of the reinforcing efficiency of clay in CPN to the imperfect exfoliation of clay. This idea was successfully applied for storage modulus and coefficient of thermal expansion in clay/epoxy nanocomposites [13, 18].

The analysis of pseudoparticles properties can reveal how the intercalated layers affect the behavior of CPN. The effects of pseudoparticle properties on the different moduli of CPN have not been investigated, yet. In this paper, the shear, bulk, and Young’s moduli of CPN containing pseudoparticles are studied by Norris model. Several experimental data are applied to show the correctness of the model and calculate the properties of pseudoparticles. Also, the roles of the effective parameters and the variation levels of all moduli at same conditions are determined.

## Methods

The pseudoparticles in CPN include the stacks of intercalated clay layers. In this regard, the aspect ratio, volume fraction, and modulus of pseudoparticles are different from the properties of individual layers. The modified number of intercalated layers in pseudoparticles [17] is calculated by

1 $$ \widehat{N}=N+\left(1-N\right)\left(\frac{\phi_f}{1-{\phi}_f}\right)\frac{s}{t} $$

where “*N*” is the number of intercalated clay layers, “*ϕ*_*f*_” is volume fraction of nanofiller, “*s*” is the distance between the intercalated clay layers, and “*t*” is the thickness of individual layers. Using Eq. 1, the aspect ratio, volume fraction, Poisson ratio, shear modulus, and bulk modulus of pseudoparticles can be expressed, respectively, by

2 $$ {\alpha}_{\mathrm{pse}}=\frac{\alpha }{\widehat{N}}\left(\frac{1}{1+\left(1-1/\widehat{N}\right)\frac{s}{t}}\right) $$

3 $$ {\phi}_{\mathrm{pse}}={\phi}_f\left[1+\left(1-1/\widehat{N}\right)\frac{s}{t}\right] $$

4 $$ {\upsilon}_{\mathrm{pse}}={\upsilon}_f\left[\frac{1}{1+\left(1-1/\widehat{N}\right)\frac{s}{t}}\right]+{\upsilon}_m\left[\frac{\left(1-1/\widehat{N}\right)\frac{s}{t}}{1+\left(1-1/\widehat{N}\right)\frac{s}{t}}\right] $$

5 $$ {G}_{\mathrm{pse}}={G}_f\left[\frac{1}{1+\left(1-1/\widehat{N}\right)\frac{s}{t}}\right]+{G}_m\left[\frac{\left(1-1/\widehat{N}\right)\frac{s}{t}}{1+\left(1-1/\widehat{N}\right)\frac{s}{t}}\right] $$

6 $$ {K}_{\mathrm{pse}}={K}_f\left[\frac{1}{1+\left(1-1/\widehat{N}\right)\frac{s}{t}}\right]+{K}_m\left[\frac{\left(1-1/\widehat{N}\right)\frac{s}{t}}{1+\left(1-1/\widehat{N}\right)\frac{s}{t}}\right] $$

where “*α*,” “*ν*_*f*_,” “*G*_*f*_,” and “*K*_*f*_” are the aspect ratio, Poisson ratio, shear modulus, and bulk modulus of individual layers, respectively. “*α*” is defined as *l*/*t*, where “*l*” is the length/diameter of layers. Also, “*ν*_*m*_,” “*G*_*m*_,” and “*K*_*m*_” are the Poisson ratio, shear modulus, and bulk modulus of polymer matrix in that order.

Norris [19] suggested a model for the shear and bulk moduli of composites reinforced by very thin oblate spheroids as

7 $$ G={G}_m+\frac{1}{15}{\phi}_f{\left[\frac{\pi }{8\alpha}\frac{3-4{\nu}_m}{G_m\left(1-{\nu}_m\right)}+\frac{1-{\nu}_f}{G_f\left(1+{\nu}_f\right)}\right]}^{-1}+\frac{2}{5}{\phi}_f{\left[\frac{\pi }{16\alpha}\frac{7-8{\nu}_m}{G_m\left(1-{\nu}_m\right)}+\frac{1}{G_f}\right]}^{-1} $$

8 $$ K={K}_m+\frac{4}{9}{\phi}_f{\left[\frac{\pi }{8\alpha}\frac{3-4{\nu}_m}{G_m\left(1-{\nu}_m\right)}+\frac{1-{\nu}_f}{G_f\left(1+{\nu}_f\right)}\right]}^{-1} $$

The Young’s modulus of isotropic solids such as nanocomposites containing dispersed nanoparticles [20] can be determined by

9 $$ E=\frac{9KG}{3K+G} $$

In this study, the experimental Young’s modulus of CPN is compared with the calculation of Eq. 9 as the prediction of Norris model. Moreover, a relative modulus can be defined as the modulus of nanocomposites divided to the modulus of neat matrix, for example, relative shear modulus is *G*/*G*_*m*_. To evaluate the influences of the pseudoparticles on the moduli of nanocomposites, the aspect ratio, volume fraction, Poisson ratio, and shear and bulk moduli of pseudoparticles are considered in the Norris model.

## Results and Discussion

The properties of pseudoparticles are applied in Norris model to study the effects of intercalated layers on the shear, bulk and Young’s moduli of polymer nanocomposites. In this regard, firstly the models are compared with the experimental data of some samples. Afterwards, the effects of main parameters on the predicted moduli are investigated.

**Fig. 2:**
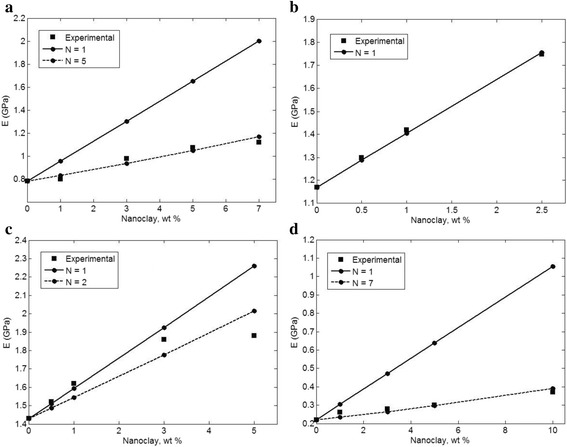
The experimental data and model predictions at different “*N*” for **a** organoclay (modified clay)/PP [10], **b** Cloisite 30B/PA12 [21], **c** unmodified clay/PP [11], and **d** organoclay/PCL [22] samples

Figure 1 illustrates the experimental measurements of Young’s modulus as well as the calculations of Eq. 9 assuming the pseudoparticles. When a complete exfoliation of clay layers (*N* = 1) is assumed in the polymer matrices, the Norris model overpredicts the Young’s modulus in most samples as expected. But, when the pseudoparticles are taken into account in the samples, the calculations show a good agreement with the experimental data at all filler concentrations. The calculations fit with the experimental data at *N* = 5, 1, 2, and 7 in organoclay (modified clay)/PP/from [10], Cloisite 30B/PA12 from [21], unmodified clay/PP from [11], and organoclay/PCL from [22]. Accordingly, the Norris model can suggest acceptable predictions for CPN assuming the pseudoparticles. Also, it is found that only PA12/Cloisite 30B sample contains the exfoliated layers and others include the pseudoparticles of clay layers. The number of layers in the pseudoparticles (*N*) is different in the reported samples indicating the dissimilar levels of reinforcement. According to Fig. 2, the exfoliated layers of clay cause a higher reinforcement in the samples compared to intercalated layers in the pseudoparticles as mentioned before. So, much effort should be made to exfoliate the clay layers in the polymer matrix by some techniques such as compatibilizing, functionalizing, and surface modification [23–25].

**Fig. 3:**
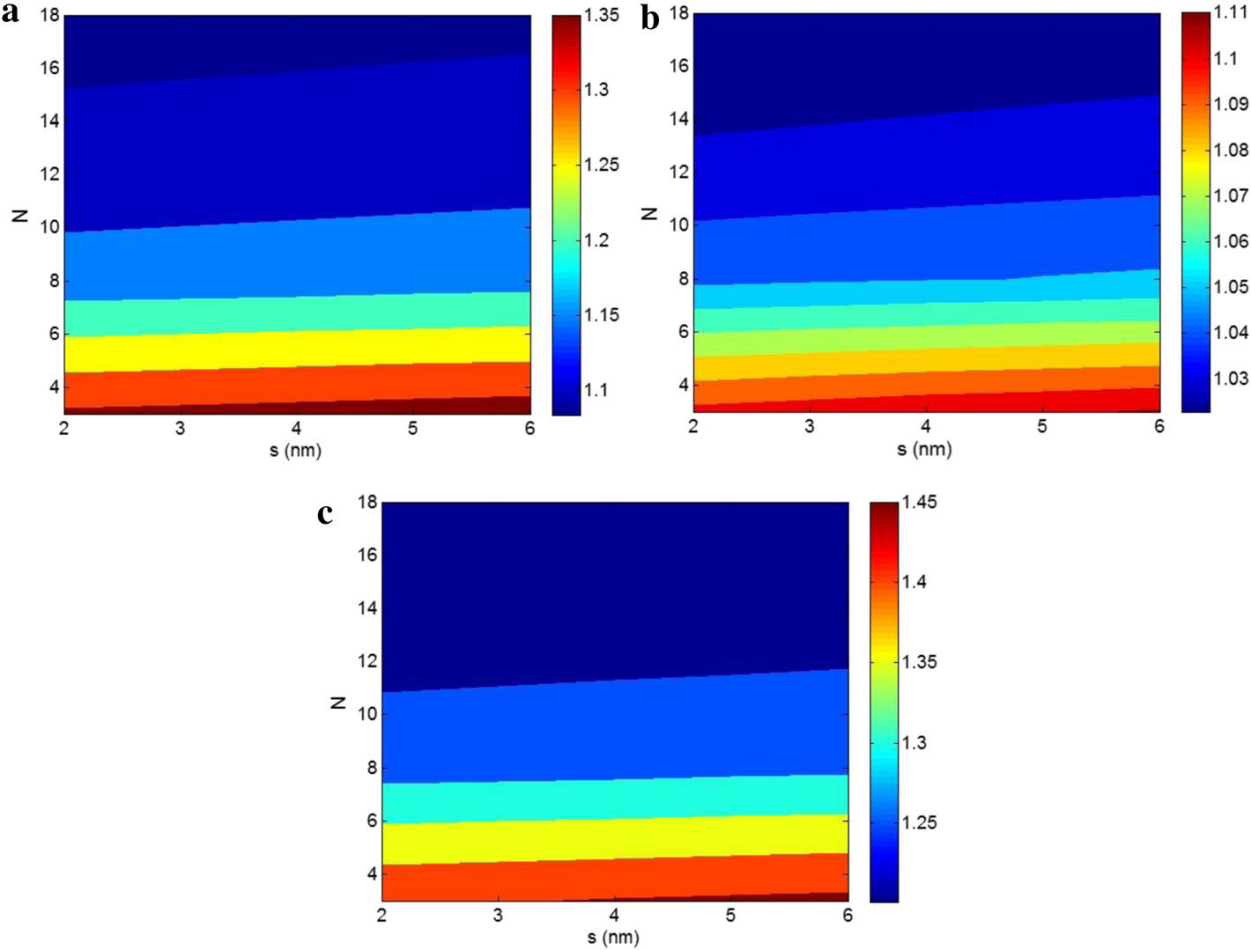
The effects of “*s*” and “*N*” parameters on the different relative moduli of CPN containing pseudoparticles with *t* = 1 nm, *ϕ*_*f*_ = 0.02, *E*_*m*_ = 2 GPa, α = 200, *ν*_*m*_ = 0.4, and *ν*_*f*_ = 0.2: **a** shear, **b** bulk, and **c** Young’s moduli

Figure 3 shows the contour plots for dependance of relative shear, bulk, and Young’s moduli to “*s*” and “*N*” parameters at *t* = 1 nm, *ϕ*_*f*_ = 0.02, *E*_*m*_ = 2 GPa, *α* = 200, *ν*_*m*_ = 0.4, and *ν*_*f*_ = 0.2. It is found that the “*s*” parameter cannot significantly change the levels of all moduli, whereas the “*N*” factor causes different moduli in the CPN. A low “*N*” presents high moduli which shows the negative effects of this parameter on the moduli of nanocomposites. It means that the large number of clay layers in the pseudoparticles decreases the moduli of samples. Accordingly, the high content of intercalated clay causes a low reinforcement compared to the exfoliated one.

As observed in Fig. 3, the relative shear, bulk, and Young’s moduli reach to 1.35, 1.11, and 1.45 at the best condition. So, the “*s*” and “*N*” parameters induce the highest range of variation in Young’s modulus, while the bulk modulus shows the smallest change among all moduli at the same conditions.

**Fig. 4:**
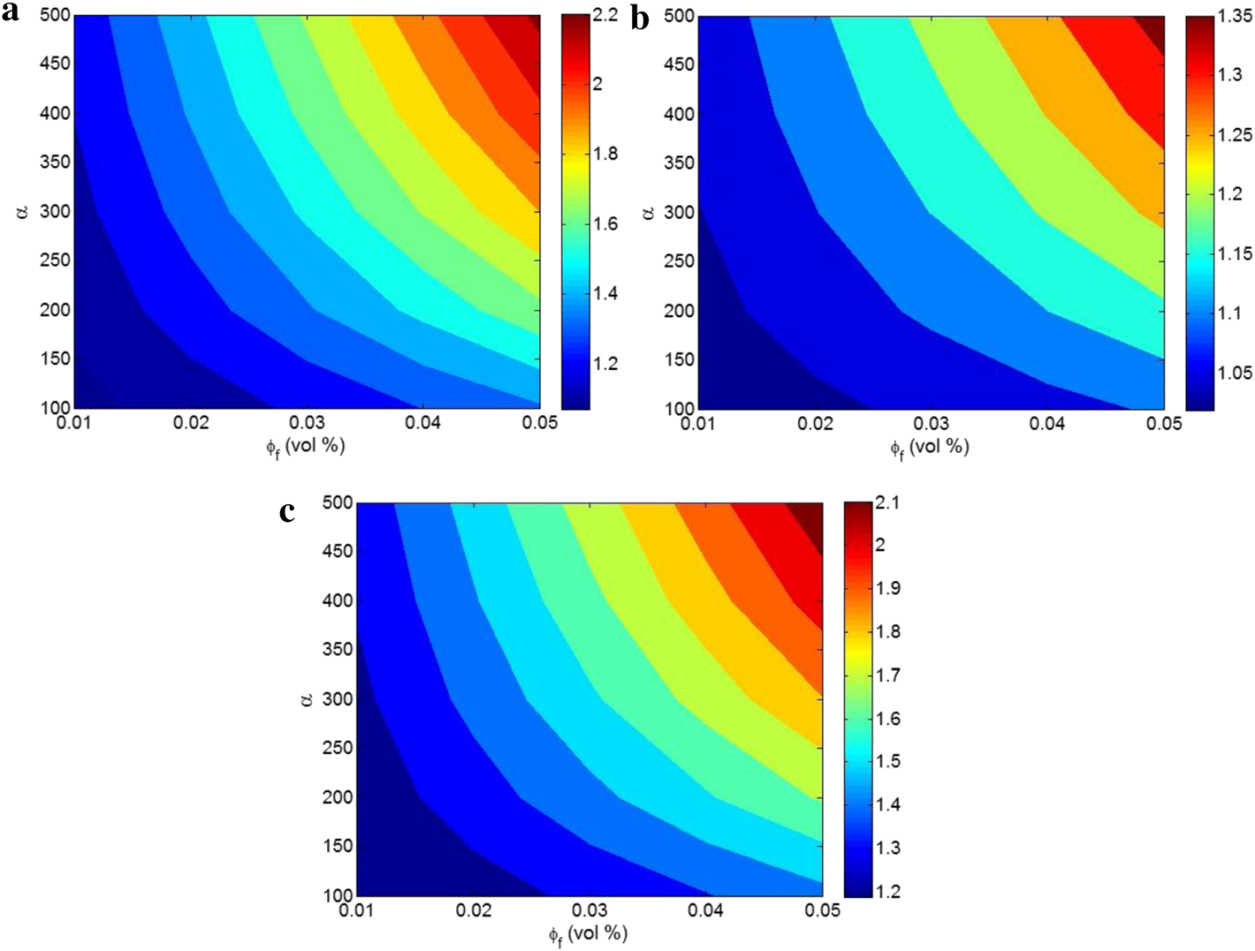
Contour plots for dependence of relative moduli to “*ϕ*_*f*_” and “*α*” at *t* = 1 nm, *s* = 3 nm, *N* = 5, *E*_*m*_ = 2 GPa, *ν*_*m*_ = 0.4, and *ν*_*f*_ = 0.2: **a** shear, **b** bulk, and **c** Young’s moduli

Figure 4 illustrates the effects of “*ϕ*_*f*_” and “*α*” on the different relative moduli of CPN containing pseudoparticles at t = 1 nm, s = 3 nm, N = 5, E_m_ = 2 GPa, ν_m_ = 0.4 and ν_f_ = 0.2. As observed, the best levels of all moduli are obtained by the highest values of “*ϕ*_*f*_” and “*α*.” Therefore, these parameters cause a direct link with the moduli of CPN containing pseudoparticles. A higher number of clay layers in the polymer matrix introduces a greater reinforcement in CPN, due to the natural stiffening effect of clay layers with *E*_*f*_ = 180 GPa. As a result, it is logical that a high content of clay layers makes a significant reinforcement in polymer nanocomposite. Also, a large aspect ratio of layers generally causes a high stiffness in nanocomposites, due to the high interfacial area between polymer matrix and nanoparticles which efficiently transfers the stress from polymer matrix to nanoparticles [26–28]. As a result, the pseudoparticles do not change the dependance of moduli to “*ϕ*_*f*_” and “*α*” parameters. However, the relative shear, bulk and Young’s moduli reach to 2.2, 1.35, and 2.1 at the highest levels of “*ϕ*_*f*_” and “*α*” as illustrated in Fig. 4. Accordingly, the highest variation is observed for shear modulus, while the bulk modulus shows the smallest range of variation.

**Fig. 5:**
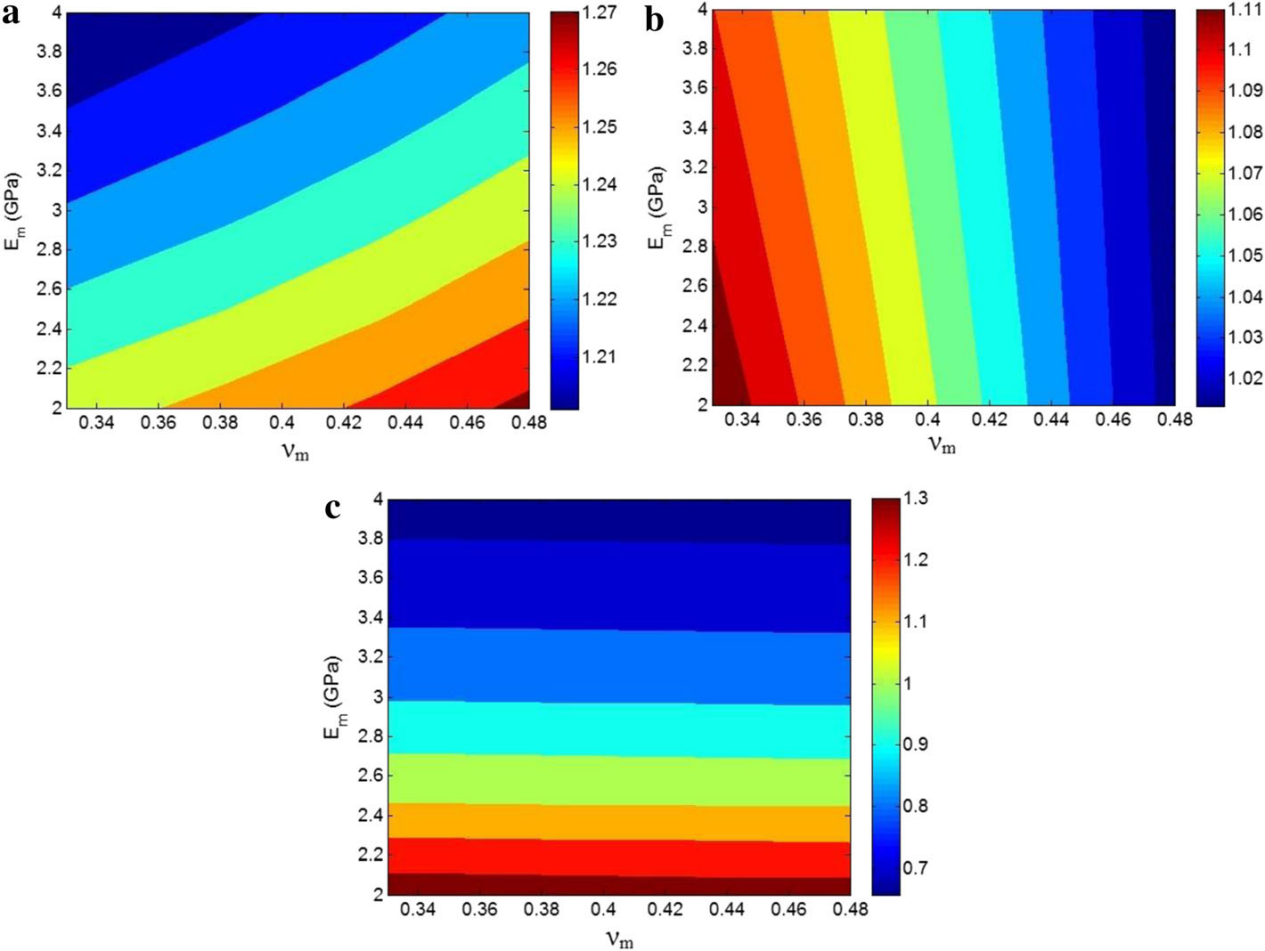
Relative **a** shear, **b** bulk, and **c** Young’s moduli of CPN containing pseudoparticles as a function of “*ν*_*m*_” and “*E*_*m*_” at *ϕ*_*f*_ = 0.02, *t* = 1 nm, *s* = 3 nm, *N* = 5, *α* = 200, and *ν*_*f*_ = 0.2

Figure 5 also demonstrates the relative moduli of CPN filling with pseudoparticles as a function of “*ν*_*m*_” and “*E*_*m*_” at *ϕ*_*f*_ = 0.02, *t* = 1 nm, s = 3 nm, *N* = 5, *α* = 200, and *ν*_*f*_ = 0.2. It is clearly shown that the “*ν*_*m*_” and “*E*_*m*_” factors differently affect the moduli of CPN by Norris model at the same levels of other parameters. The shear modulus shows the high levels at the large “*ν*_*m*_” and small “*E*_*m*_.” The maximum relative shear modulus of 1.27 is obtained by *ν*_*m*_ = 0.48 and *E*_*m*_ = 2 GPa. However, the minimum relative shear modulus of 1.2 is reported at ν_m_ = 0.33 and E_m_ = 4 demonstrating that the shear modulus decreases by reduction in “*ν*_*m*_” and increase in “*E*_*m*_.” As observed in Fig. 5a, the shear modulus does not considerably change by these parameters.

It can be also said that the bulk modulus only depends to the level of “*ν*_*m*_” and the “*E*_*m*_” parameter does not play an important role in the “*K*.” A high “*K*” is obtained by a small “*ν*_*m*_” and an increasing in “*ν*_*m*_” reduces its level. This observation indicates the inverse relation between the bulk modulus of CPN and Poisson ratio of polymer matrix. The bulk modulus also does not mainly vary by “*ν*_*m*_” and “*E*_*m*_” factors. According to Fig. 5c, the Young’s modulus only relates to “*E*_*m*_” parameter and “*ν*_*m*_” cannot play a role in its ranges. A high value of “*E*_*m*_” produces a low relative “*E*” at different levels of “*ν*_*m*_.” The low improvement of Young’s modulus by high “*E*_*m*_” may be related to the low difference between the Young’s moduli of polymer matrix and nanoparticles in this condition. Moreover, the low range of Poisson ratio of polymers from 0.33 to 0.5 cannot significantly affect the moduli of CPN. Despite the shear and bulk moduli, the Young’s modulus displays large variation by “*ν*_*m*_” and “*E*_*m*_” parameters.

Generally, the shear and Young’s moduli of CPN containing pseudoparticles show a high change by the properties of pseudoparticles, while the bulk modulus demonstrates a negligible difference at different levels of whole parameters.

## Conclusions

The effects of the pseudoparticles characteristics on the shear, bulk, and Young’s moduli of CPN were studied by Norris model. When the complete exfoliation of clay layers (*N* = 1) was supposed in CPN, the model overpredicts the modulus in most samples. But, assuming the pseudoparticles produces a good agreement between the calculations and the experimental data at all filler concentrations. The calculations well fit with the experimental data at different values of “*N*” as a sign of dissimilar reinforcement in the samples. It was found that “*s*” parameter cannot significantly change the levels of moduli, whereas the “*N*” parameter causes different moduli in the nanocomposites. A low “*N*” presents high moduli which shows that the large number of clay layers in the pseudoparticles decrease the moduli of samples. The best levels of all moduli were also obtained by the highest “*ϕ*_*f*_” and “*α*,” demonstrating a direct link between these parameters and the moduli of CPN containing pseudoparticles. Additionally, the properties of polymer matrix, “*ν*_*m*_” and “*E*_*m*_” parameters played different roles in the shear, bulk, and Young’s moduli of CPN. Generally, the shear and Young’s moduli of CPN showed a high range of change by the properties of pseudoparticles, while the bulk modulus established a negligible difference at different levels of all factors.
